# Spatio-temporal distribution and habitat preference of necrophagous Calliphoridae based on 160 real cases from Switzerland

**DOI:** 10.1007/s00414-021-02769-8

**Published:** 2022-01-22

**Authors:** Jiri Hodecek, Pavel Jakubec

**Affiliations:** 1 Swiss Human Institute of Forensic Taphonomy, University Centre of Legal Medicine, CH-1000 Lausanne, Switzerland; 2 Musée Cantonal de Zoologie, Palais de Rumine, Place de la Riponne 6, CH-1014 Lausanne, Switzerland; 3 Department of Ecology, Faculty of Environmental Sciences, Czech University of Life Sciences Prague, Kamýcká 129, CZ-165 00 Praha – Suchdol, Czech Republic

**Keywords:** Blowflies, Switzerland, Altitude, Real cases, Investigation, Forensic entomology

## Abstract

Necrophagous blowflies (Diptera: Calliphoridae) are of great importance particularly during investigations of suspicious deaths. Many studies have analyzed the distribution of blowflies based on pig experiments and baited trapping; however, data from real case scenarios are rarely used. In this article, the distribution of blowflies found during investigations of 160 real cases during 1993–2007 in Switzerland is evaluated based on habitat, altitude, and season. Ten species of blowflies were present in 145 out of the 160 cases. The most common species was *Calliphora vicina*, which occurs throughout the year and was present in 69 % of all cases. *Lucilia sericata*, *Calliphora vomitoria*, and *L. caesar* were identified among the rest of the flies as species of great forensic importance mainly due to their distributional patterns. After a comparison with a similar dataset from Frankfurt, Germany, some surprising differences were determined and discussed. The biggest discrepancies between our dataset and the German dataset were in the occurrences of *L. sericata* (30 % vs. 86 %, respectively), *Phormia regina* (5 % vs. 43 %), and *L. ampullacea* (1 % vs. 45 %). The life-history strategies and intraspecific behavioral variability of blowflies remain understudied, although they can be essential for an unbiased approach during a death investigation. Further research and comparison of occurrence patterns across the area of distribution of blowflies are therefore needed and recommended.

## Introduction

The blowflies belong to the most important family of necrophagous Diptera used during forensic investigations. Species of this family are usually common, highly abundant and among the first colonizers of a dead body [1, 2]. Their presence is mainly determined by the season (i.e., the annual phenology of particular species), habitat type (within the area of distribution of the species) and by the ambient temperature and microclimatic conditions at the death scene (i.e., the requirements of particular species to finish their life cycle) [3]. Therefore, every forensic entomologist needs to not only identify the focal species, but also to know their local spatio-temporal distribution and habitat preferences. This is particularly important in the case of the blowflies, as they are usually cosmopolitan species with not always consistent habitat association patterns. The patterns can change geographically depending on the local offer of the habitat types [4]. An example of such species is *L. sericata*, which can locally be a common species of open pastures [5, 6], while at the same time it is a common inhabitant of urban areas (often found in indoor cases) [7–9].

The common design of experiments, which aims to map the distribution and habitat preference of blowflies, often counts on baited traps usage (bottle traps, inverted cone traps, Schoenly traps) which attract the specimens by using different kinds of attractants [4, 10–14]. The bottle traps certainly can serve as a basic tool for monitoring of blowflies; however, the attractiveness depends on the type of bait used. Furthermore, such traps can induce biases, because they can be neglected or even avoided by certain species due to their preferred diet [3, 15–17] and care should be taken when extrapolating such results to case scenarios [18, 19]. For example, *C. vomitoria* and *Protophormia terraenovae* are common blowflies, which can be often found on human corpses; however, they might avoid bottle traps, because they prefer a cadaver of a certain size [5, 20, 21]. These specific preferences of certain species can potentially bias such research and result in an under- or overestimation of abundance or even the presence of some of the focal species. Several studies focused on the distribution of blowflies using pig cadavers have already been published [21–24] and while such an approach is certainly more suitable (with pigs to be the most accurate option compared to human cadavers), there have not been many studies using the data of real cases to obtain such results [3, 25–27]. The reasons are probably (1) the size and time needed for obtaining a large enough dataset for making meaningful conclusions and (2) the legal complications stemming from the publication of such a sensitive dataset in some countries.

The data from real cases collected over long periods of time (ideally decades) are rarely published. Such dataset can however be extremely important for understanding the local fauna linked to real case scenarios in different geographic locations especially when forensic entomologists rely upon old and restricted taxonomic keys, which may not include all the local species [19]. The real case data are also irreplaceable for elucidation of the ecology, colonization patterns and spatio-temporal distribution of blowflies for indoor cases. Indoor cases represent a large portion of the casework during medico-legal investigations [3, 19, 26, 27]. Furthermore, indoor habitats have a completely different thermal dynamic in comparison to outdoor habitats and this dynamic differs based on local climatic conditions [19, 22]. Understanding local climatic conditions and how species at different habitats adapt to them can provide crucial information during investigations and the post-mortem interval (PMI_min_) estimations. However, it is nearly impossible to conduct such experimental study under controlled conditions due to possible health risk issues and financial constraints; therefore, the real case data represent a unique window allowing a closer look at the life of blowflies under such settings. In this article, an extensive dataset of blowflies built from 160 real cases over a period of 14 years (1993–2007) in Switzerland has been analyzed, along with their spatio-temporal distribution and habitat preference. This study enables comparisons with other forensic and ecological studies, particularly with respect to larger scale trends in insect colonization patterns.

## Material and methods

**Fig. 1:**
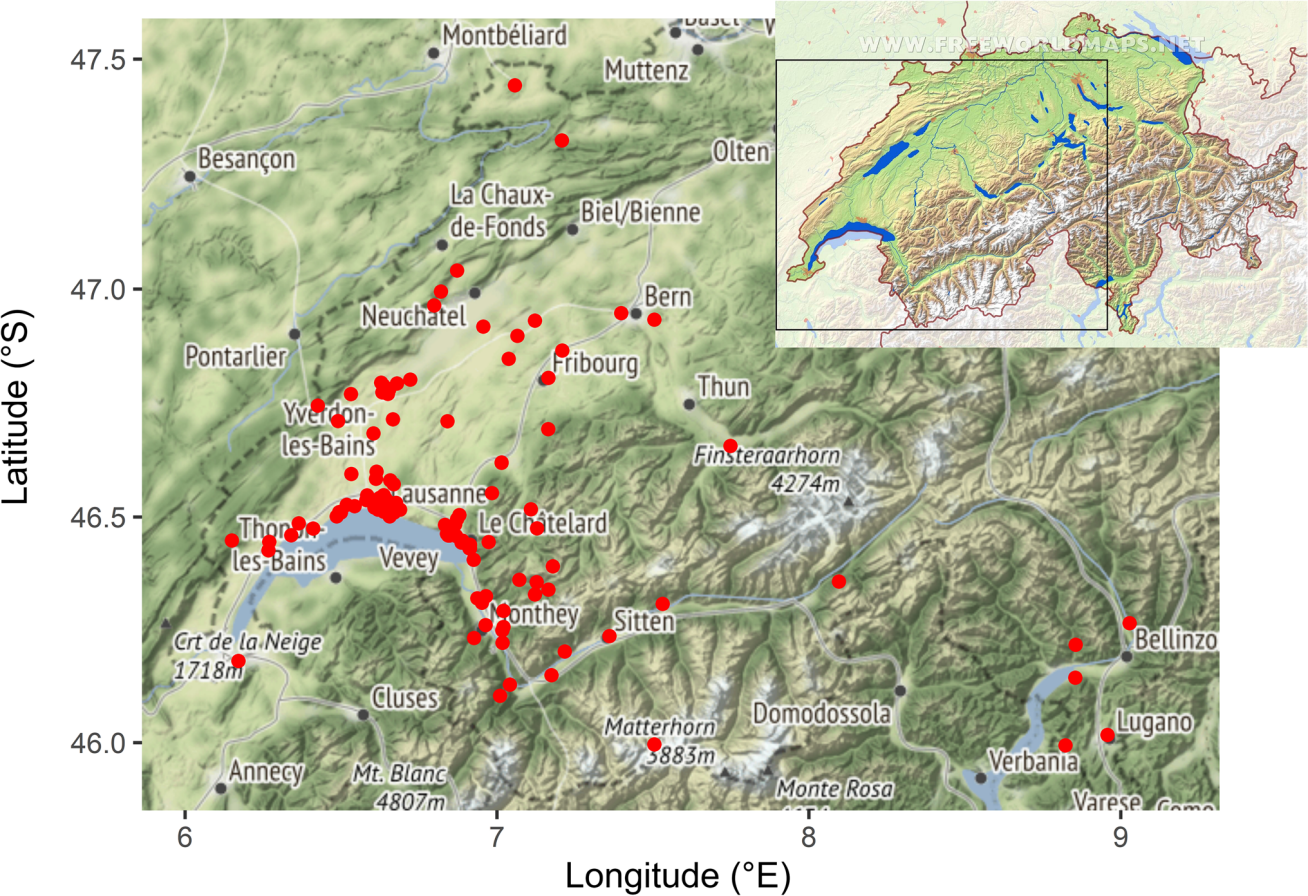
The map of real case investigations in and around Lausanne during 1993–2007 (the map of Switzerland downloaded from www.freeworldmaps.net)

The data comes from 160 criminal investigations involving entomological expertise, which were conducted in between the years of 1993–2007 in and around Lausanne in Switzerland [28]. The area includes canton of Vaud, canton of Geneva, canton of Fribourg, canton of Valais, and canton of Ticino (Fig. 1).

The insects were collected by Claude Wyss, who was a forensic investigator and an enlightened amateur in forensic entomology, working for the police in the 1990s and early 2000s. The collection was performed mostly at the death scene, but sometimes also during the autopsy. The real proportion of the scene/autopsy sampling is not known. The sampling techniques used to collect the insect material are described in Cherix et al. (2012) [29]. Immature stages of blowflies (Calliphoridae) collected on corpses were reared in the laboratory under controlled conditions to the adult stage and later used for the post-mortem interval (PMI_min_) estimations. All collected insect material was mounted and stored in the Museum of Zoology in Lausanne. The blowflies were originally identified by Wyss, Cherix, and Faucherre based on their terminalia and relying on a combination of the identification keys and the reference collection at the Zoological Museum, University of Copenhagen [29]. The material was revised in 2019–2020 by Hodecek, who worked on the correction and actualization of the database (as many identifications were either wrong or missing). The revision of the family Calliphoridae was made based on the actualized available key by Szpila [30].

### Data management and statistical evaluation

Species from family Calliphoridae with more than 5 records were selected for the analyses (i.e., *Cynomia mortuorum* with 2 records and *Lucilia ampullacea* with one record were excluded). We also made sure that the information regarding the date of discovery (month, year), habitat where the body was found, and an altitude were available. We worked only with living developmental stages, i.e., we did not include puparia or dead adults in our analyses. Unfortunately, we did not have information about the sex and age of the deceased, manner of death, and the PMI_min_ for all the cases.

**Fig. 2:**
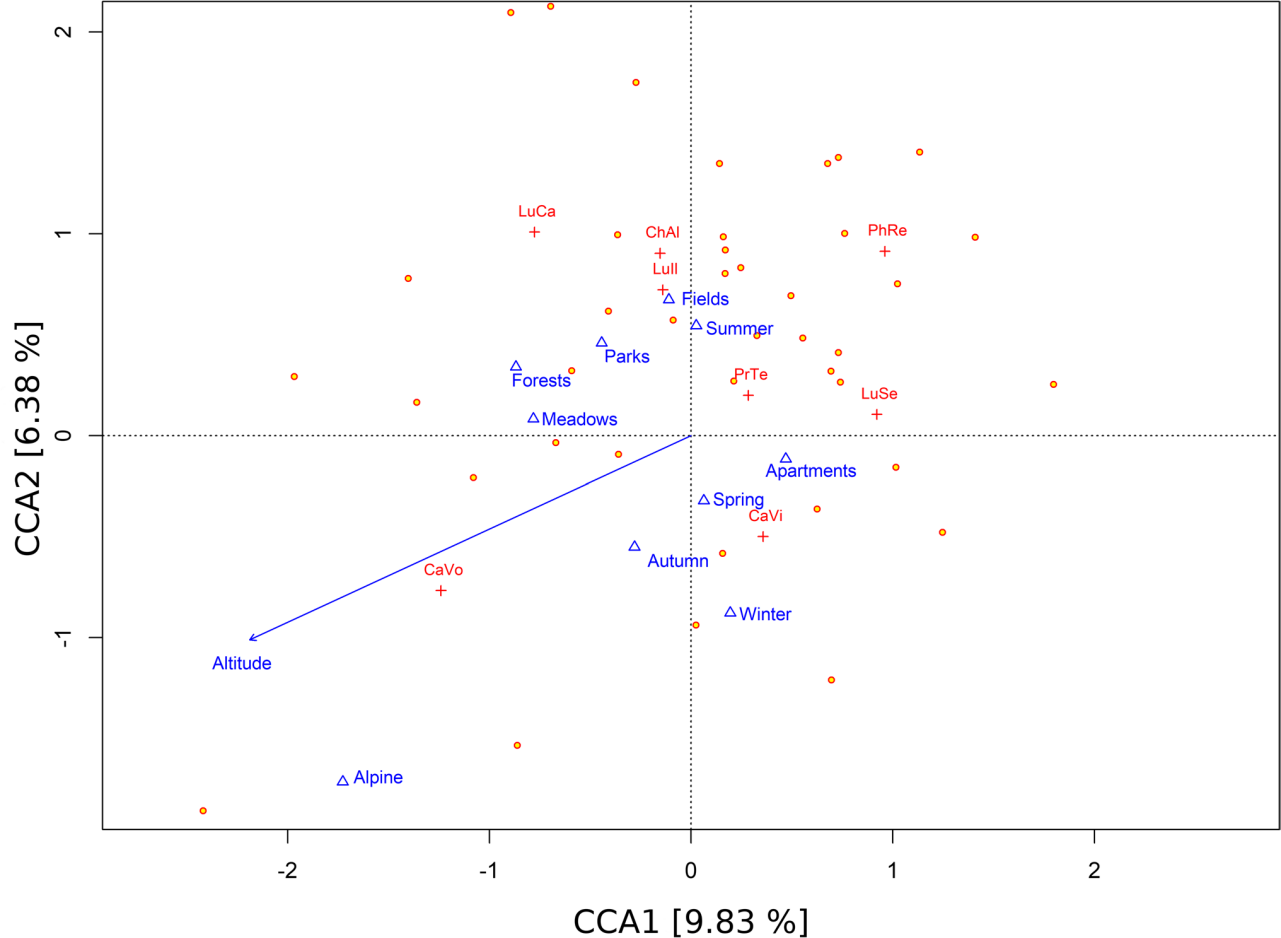
CCA ordination triplot, cases are represented by circles, species by crosses and factors as triangles. Species names were abbreviated (*Calliphora vicina* (CaVi), *Calliphora vomitoria* (CaVo), *Chrysomya albiceps* (ChAl), *Lucilia caesar* (LuCa), *Lucilia illustris* (LuIl), *Lucilia sericata* (LuSe), *Phormia regina* (PhRe), *Protophormia terraenovae* (PrTe)). Amount of explained variability represented by CCA axes is given in square brackets

The original database we worked with contained a detailed description of the biotope for 115 cases. We were able to obtain the information about the habitats for the missing 45 cases thanks to the coordinates of the body localizations, which—altogether with public accessible historical satellite images of the places from Google Earth Pro (version 7.3.3.7786) [31]—gave us the basic type of the biotope necessary for our analyses. All the cases were divided into 6 biotope types—*forest* (all types), *meadow* (including pastures and grasslands), *field* (all types of agricultural areas such as vineyards or cornfields), *alpine* (high mountain area without vegetation), *park* (including gardens), and *apartment* (all indoor cases). The types of the habitat were then used as variables for canonical correspondence analysis (CCA) (Fig. 2).

In order to visualize and test the relationships between the composition of the communities associated with human remains across continuous explanatory factor altitude and two discrete factors of season and habitat, the constrained CCA from R package vegan was used [32]. The significance of each environmental variable was tested by permutation tests with 9999 permutations to establish if the relationship was significantly affected by the factors in question, using anova.cca function from the package vegan as well [32].

The effect of altitude on the presence or absence of focal species was established using a binomial linear regression analysis with mixed effects (GLMM). The response variable was the presence/absence of the species and fixed effect explanatory variables were species’ identity and the natural logarithm of altitude. As the random effect explanatory variable, we used months, to eliminate the season’s effect. Furthermore, intercept and regression slope were allowed to vary between species, so they could be evaluated independently.

The significance levels in all analyses were set at 5%. Data management and statistical evaluation were performed in R (version 4.0.3.) [33].

## Results

**Fig. 3:**
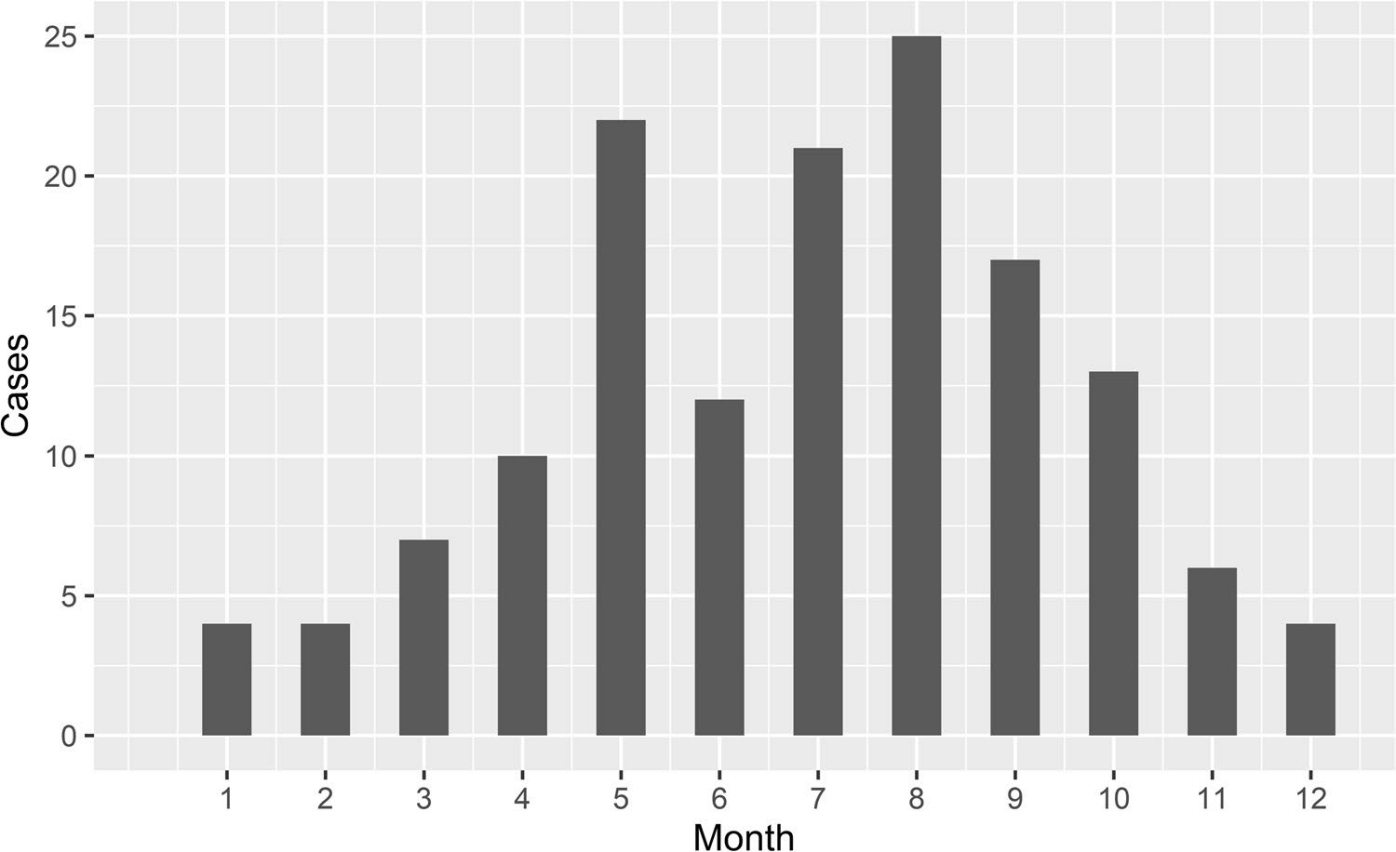
Histogram of monthly distribution of real case investigations during 1993–2007

Blowflies were present in 145 out of 160 real case investigations of dead bodies in our dataset, which is in concordance with the widespread image of blowflies being the most important family of necrophagous flies used for criminal investigations [1, 2]. There was a clear pattern of seasonal distribution of the cases with a peak in summer and a gap in winter months (Fig. 3). Out of the 145 cases, 88 (i.e., 61 %) were located indoor. The most dominant species was *C. vicina* (100 cases—69 %), followed by *L. sericata* (44 cases—30 %), *C. vomitoria* (42 cases—29 %), and *L. caesar* (39 cases—27 %). In total, there were 10 species of blowflies present on the bodies (Table 1).

**Table 1:** The comparison of the species occurrence in real case investigations in Lausanne (1993–2007) and Frankfurt (2014–2016) (expressed as a percentage of occurrences)

Species	N. of cases (Hodecek & Jakubec)	Percentage (Hodecek & Jakubec)	N. of cases (Bernhardt et al.)	Percentage (Bernhardt et al.)
*Calliphora vicina*	100	68.97	22	43.14
*Lucilia sericata*	44	30.34	44	86.27
*Calliphora vomitoria*	42	28.97	12	23.53
*Lucilia caesar*	39	26.90	10	19.61
*Lucilia illustris*	23	15.86		
*Protophormia terrae-novae*	21	14.48	16	31.37
*Chrysomya albiceps*	13	8.97	5	9.80
*Phormia regina*	7	4.83	22	43.14
*Cynomya mortuorum*	2	1.38	0	0.00
*Lucilia ampullacea*	1	0.69	23	45.10
**Total cases**	**145**		**51**	

In Bernhardt et al. (2018) comparative study, *L. caesar* and *L. illustris* were not differentiated

**Fig. 4:**
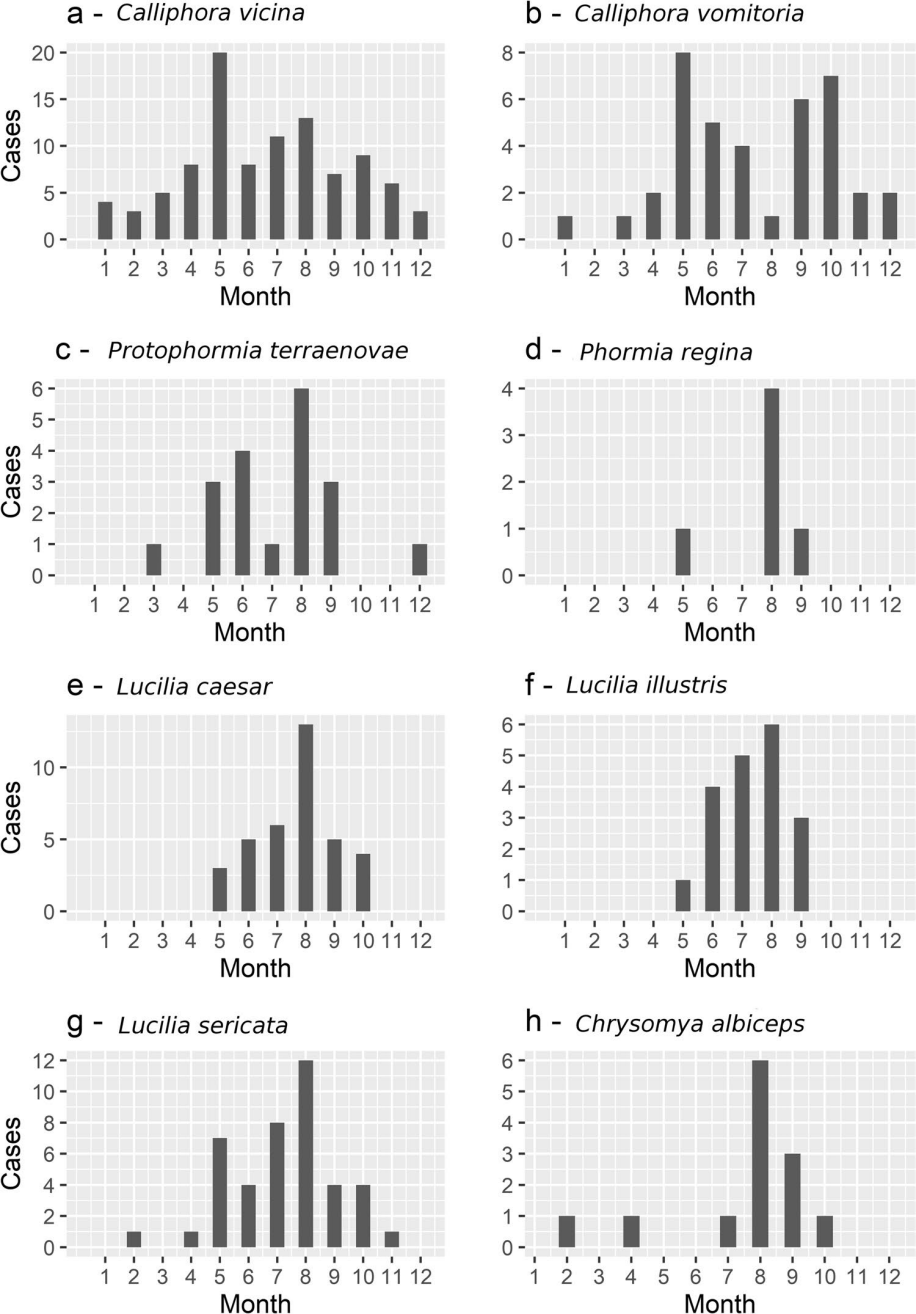
**a**–**h** Histograms depicting the monthly distribution of records of occurrence of all stages for each species of family Calliphoridae (species with less than 5 records were excluded)

The CCA analysis was able to explain 19.98 % of the total variability (0.534 out of 2.672 inertia value) while first (inertia = 0.263) and second (0.170) canonical axes were responsible for explaining 9.834 and 6.375 % of total variation respectively. Its results show that all environmental factors (altitude, season, and habitat type) prove to be significantly associated with the occurrences of observed blowflies (*p*-value < 0.001). Specifically, altitude (*F* value = 8.382; Df = 1; *p* value < 0.001), season (*F* value = 3.312; Df = 3; *p* value < 0.001) and habitat type (*F* value = 3.377; Df = 5; *p* value < 0.001). *Cynomia vomitoria* shows a high correlation with the increasing altitude and also with colder parts of the year (autumn and winter) (Fig. 2). This can be further seen in Fig. 4b as the second peak of activity occurs in September and October and the species was observed also in all winter months except February.

**Fig. 5:**
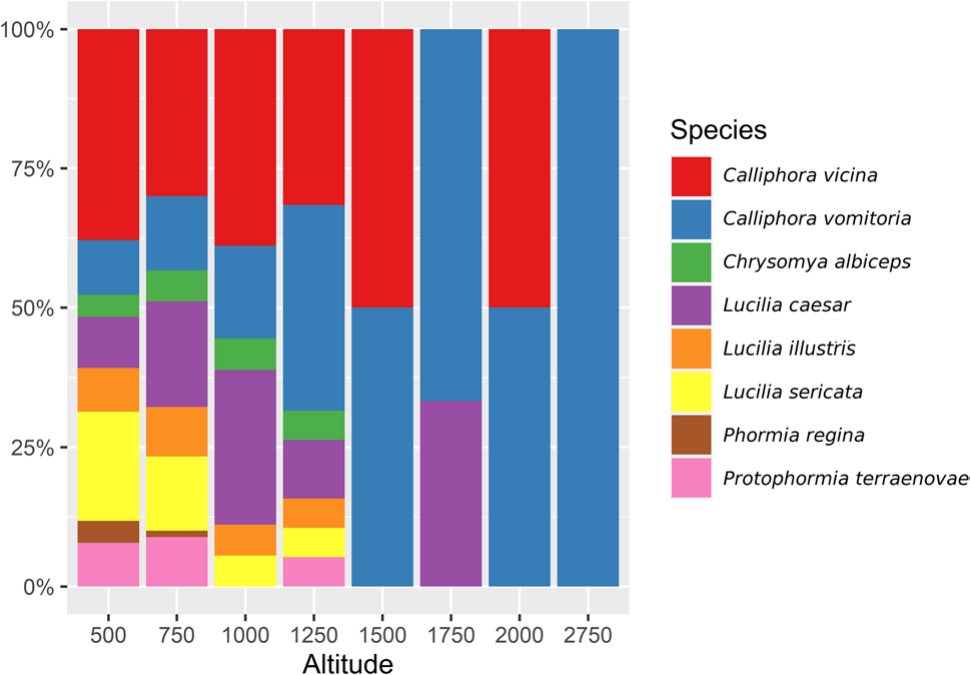
Species composition of Calliphoridae at different altitudinal intervals (expressed as a percentage of occurrences)

**Fig. 6:**
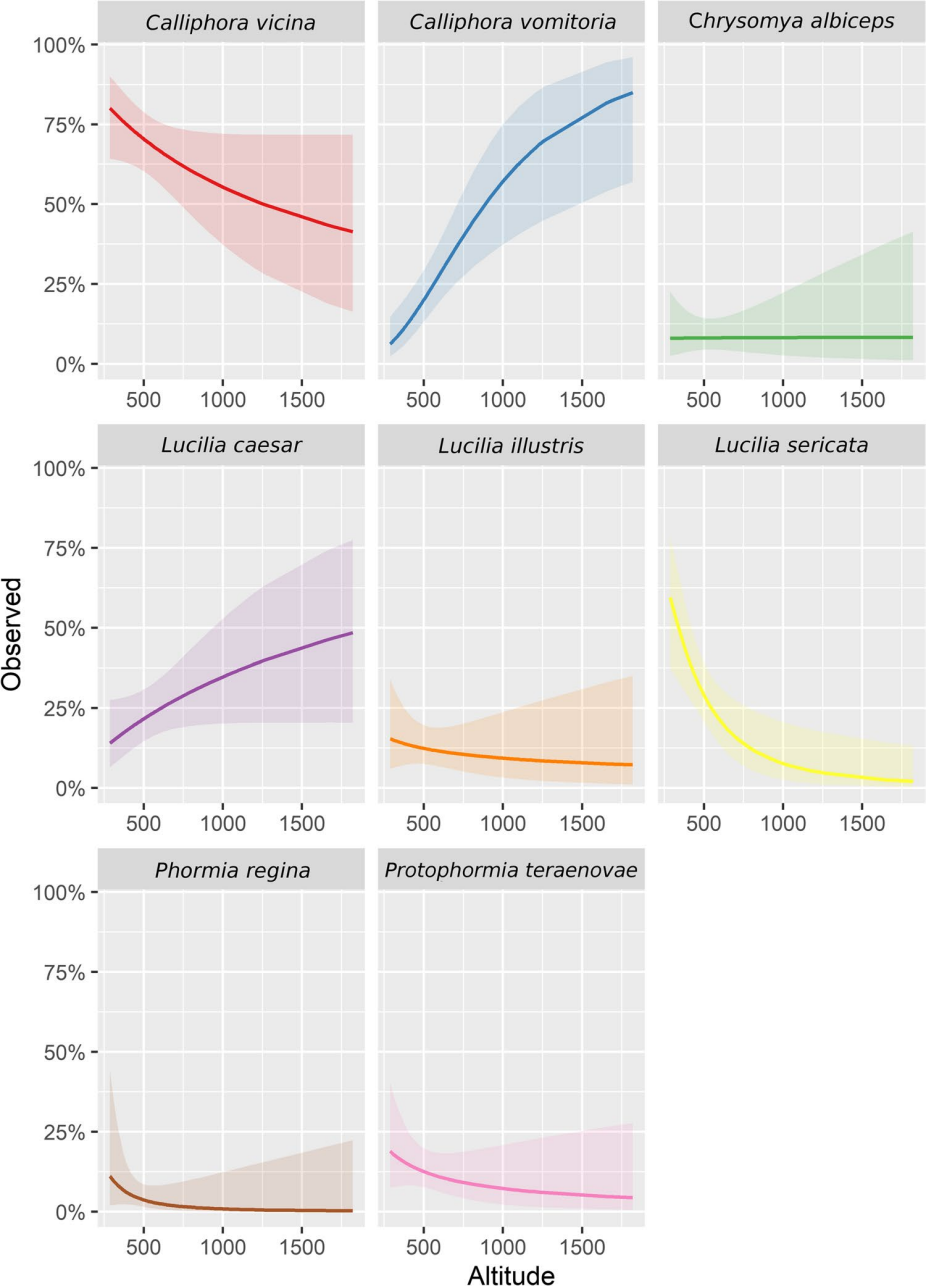
Prediction of the mean probability of occurrence (0–100) with standard errors across altitudes based on GLMM binomial model for all analyzed species

**Fig. 7:**
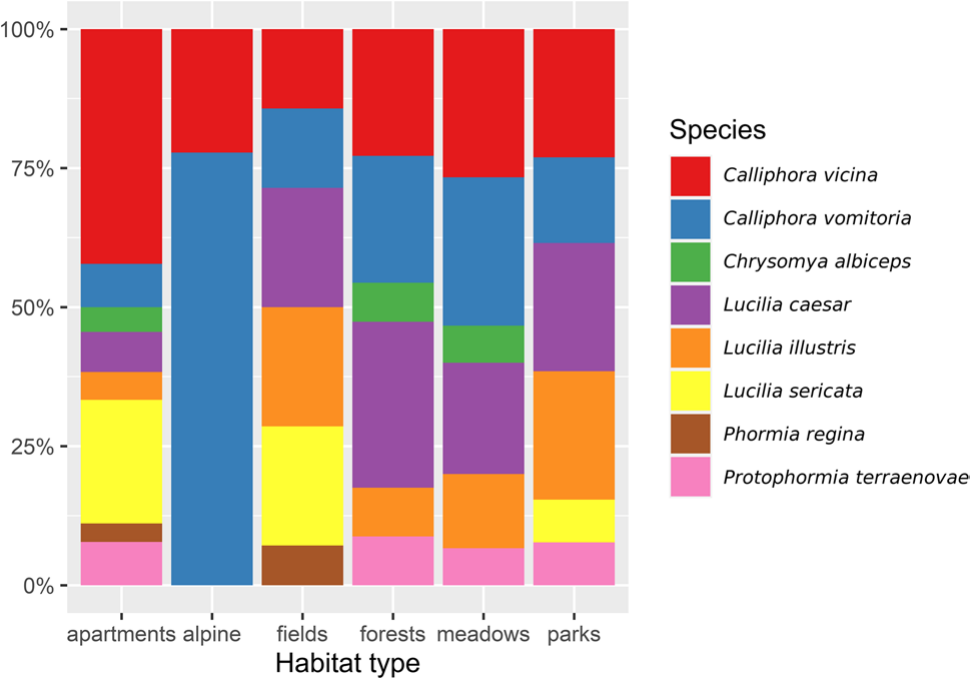
Species composition of Calliphoridae in each recognized habitat (expressed as a percentage of occurrences)

GLMM analysis showed a significant change of presence with altitude only in the case of *C. vomitoria* (*Z* value = 4.203, *p* value < 0.001) and *L. caesar* (*Z* value = 2.551, p-value = 0.011); however, more species seemed to be affected by altitude (Figs. 5 and 6). *Calliphora vicina* was the only other species correlated with the cold seasons and partially also higher altitudes (Figs. 2, 5, and 6). While *C. vomitoria* preferred the natural environment, *C. vicina* was mostly species of indoor cases and it could be found throughout the whole year (Figs. 4a and 7). The second-most dominant species—*L. sericata*—was found regularly in anthropogenic habitats, i.e., apartments, fields, and parks (Fig. 7). However, unlike *C. vicina*, its peak activity was recorded in summer (Fig. 4g). *Protophormia terraenovae* and *P. regina* were correlated with low altitudes with the peak of their activity in August (Figs. 4c–d, 5, and 6) (*P. regina* was the least common species in our analyses with only 7 observations). *Lucilia caesar* and *L. illustris* were found in all biotopes except *alpine*; however, *L. caesar* was found mostly in natural environments with the most observations in *forest* habitat, while *L. illustris* correlated with the habitat type park and field (Figs. 2 and 7). All species of the genus *Lucilia* seemed to prefer summer season and lower altitudes, except one observation of *L. caesar* at 1650 m above sea level (Figs. 4e–g, 5, and 6). *Chrysomya albiceps* was another summer species mostly active in August; however, there were 2 cases with this species present also in February and April (as live pupae) (Fig. 4h). The habitat preference of *C. albiceps* was not significant. While it was often found in environments as meadows and forests, there were some observations in the apartments as well (Fig. 7). The altitudinal distribution was similar to that of *P. terraenovae* with one occurrence at 1230 m above sea level (Figs. 5 and 6).

## Discussion

This study is the first research using a large dataset of real cases for systematic study with a focus on the spatio-temporal and altitudinal distribution of blowflies in Switzerland. All 10 species of the family Calliphoridae, which were present on the bodies, belong among common necrophagous European fauna. The most common species of our dataset was by far *C. vicina* (69 %), while in some other ecological studies from Switzerland or Germany first place often belongs to *L. sericata* [3, 12, 34]. In our case, *L. sericata* was present in 30% of cases (while still being the second-most dominant species of our dataset). The occurrence of *L. sericata* and some other focal species is surprisingly different in comparison with a similar dataset of Bernhardt et al. [3], who analyzed the Diptera assemblages of 51 human remains in the city of Frankfurt. The biggest discrepancies between the two datasets were among the following species: *L. sericata* (30 % vs. 86 %), *P. regina* (5 % vs. 43 %), and *L. ampullacea* (1 % vs. 45 %) (Table 1). Some of the reasons for such substantial differences can be the different years of sampling (1993–2007 in our case vs. 2014–2016 in Bernhardt’s dataset) or the proportion of indoor vs. outdoor cases. There were only 9 out of 51 cases (i.e., 18 %) found outdoor in Frankfurt [3], while we localized 58 out of 145 cases (i.e., 39 %) outdoor in and around Lausanne. The geographical distribution of *P. regina* and *L. ampullacea* in Europe during the years 1993–2007 could also be different from recent years due to climate change [27, 35, 36]. However, we think that the most probable reason might be the character of the local habitat composition and the topography of the compared areas. While Lausanne is situated 495 m above sea level surrounded by high mountains, Frankfurt is a low altitude city (112 m above sea level). If we take the altitude as a surrogate of the climate, we can assume that higher altitudes will be more suitable for cold-tolerating species like *C. vicina*, while lower altitudes fit better the thermophilous species such as *L. sericata*.

### Altitudinal gradient

Due to the local topography, the cases described herein are ranging from 287 m above sea level up to 2620 m above sea level (Fig. 5). Such a large altitudinal gradient allowed us to evaluate the ability of blowflies to breed and inhabit high mountain habitats. The knowledge of the blowfly distribution along the altitudinal gradient can be an important information as it can contribute to the understanding of the geographical distribution of many species as well as their local diversity [37]. There were only 3 species able to colonize human remains above 1500 m: *C. vomitoria*, *C. vicina*, and *L. caesar*. These results are in agreement with the results of [38], who studied the distribution of blowflies along an altitudinal gradient in central Spain. Baz et al. [38] determined *C. vomitoria* and *C. vicina* as species of high altitudes (most abundant in between 1600–1800 m), while *L. caesar* as a species of intermediate elevations. Unfortunately, there were only 7 bodies localized outside in high altitudes (above 1200 m). *Calliphora vomitoria* was present in all of the cases, *C. vicina* was present in 3 cases, and *L. caesar* in 1 case. Even with such a small amount of appearances in the highest altitudes, the affinity to altitude was significant for *L. caesar* and *C. vomitoria* as the trend was set in lower altitudes. *Calliphora vomitoria* is a thermophobic species [12, 39], which could explain its altitudinal preferences if we consider elevational gradient as a surrogate of climate [38]. Indeed, the temperature profile in high elevations will always be one of the limiting factors, which means that thermophilous species will visit higher altitudes only occasionally during the warmest days of the year. The occurrence of the thermophilous species in high elevations is thus also season dependent as the upper distributional limits of species may fluctuate on a seasonal or annual basis [40, 41]. All other species in our dataset preferred low elevations. The affinity to low altitudes is not surprising for thermophilous species such as *L. sericata* and *C. albiceps* (in fact, no species of *Lucilia* was able to pass 1200 m except for *L. caesar*). However, unlike *L. sericata*, *C. albiceps* is occasionally capable of reaching high elevations [38]. The highest locality where *C. albiceps* was present in our dataset was at 1230 m, where 2 living pupae were found in the forest. *Phormia regina* is the only species, which did not pass the altitude of 750 m. Our data thus suggest a strong negative correlation to altitude. However, since *P. regina* was also the least observed species of our analyses, the results of its altitudinal gradient might be incomplete. Indeed, the presence of *P. regina* was already reported as high as 2835 m on remains of a human stillborn infant [42] and up to 3566 m on rabbit carcasses [43] (both cases from Colorado). The local altitudinal distribution of this species in Switzerland should be further monitored to complete the missing data and confirm its presence in higher elevations.

### Habitat preference

The habitat preferences of all 8 analyzed species are shown in Fig. 2 as well as in Fig. 6. The results generally confirm the ecology of detected species known from previous experiments conducted in Europe. *Calliphora vicina* and *L. sericata* preferred apartments (i.e., indoor cases), which is supported by the results of many other authors [3, 44–46]. *Calliphora vicina* was present in 42 % of indoor cases, which makes it the most important species from the point of forensic investigations of indoor cases in and around Lausanne. *Lucilia sericata* was often found also in the fields or parks near human settlements. The distribution of *L. sericata* in our study is therefore mostly synanthropic, which is similar to the findings of [4, 47]. On the contrary, *C. vomitoria* is considered an indicator of natural environments [4, 47, 48] and rural sites [34]. The occurrence of *C. vomitoria* in our area was distributed among all observed habitats, with a preference for meadows, forests, and alpine habitats. Such broadscale of distribution excludes this species as a biogeographic indicator [21]. The distribution of *C. vomitoria* was partially copied by the occurrence of *L. caesar*. *Lucilia caesar* also prefers shaded locations in rural environments [12, 21, 47] and it was positively correlated with forest habitats. However, even *L. caesar* was found 13 times (15 %) in the apartments. The explanation probably is the overall majority of the indoor cases in our dataset (Table 2). As mentioned above, 61 % of all the cases were found in the apartments. All analyzed species of blowflies were found indoor (Fig. 7). *Lucilia illustris* was significantly related to parks and fields, i.e., in more synanthropic habitats than those preferred by *L. caesar*. The habitat preference and synanthropic level for *L. illustris* are somehow in between *L. sericata* and *L. caesar*. It is a heliophilic species less abundant in the shady forest localities (in comparison to *L. caesar*) as well as in the indoor cases (in comparison with *L. sericata*) [4, 12]. It has to be noted that the identification of *L. caesar* and *L. illustris* can be tricky even in adult stages, which can lead to a certain bias in results or even omittance of the species differentiation for certain analyses [3]. The non-indigenous blowfly *C. albiceps* is often described as tropical and subtropical species; however, its occurrence in Europe is already well established and it serves as a forensically important indicator for European criminal investigations for years [21, 49]. Its occurrence on human remains in and around Lausanne was rather occasional with some tendencies to prefer more natural habitats as forests and meadows. Recent data however show a bigger percentual proportion of this species on human cadavers, which can point at its increasing importance as a species of forensic importance in Switzerland (data of real cases from 2019 to 2020 in Lausanne - Hodecek unpublished). The rest of the observed species did not show any significant dependence on habitat type. This includes *P. regina* with only 7 observations and *P. terraenovae*, which was present in all habitat types except fields and alpine environment.

**Table 2:** The number of real case investigations in each habitat type during 1993–2007

Habitat type	No. of cases	Percentage
Field	7	4.83
Forest	31	21.38
Alpine	7	4.83
Meadow	7	4.83
Park	5	3.45
Apartment	88	60.69
**Total**	**145**	**100**

### Seasonal distribution

The seasonal occurrence of blowflies in Europe has already been studied in several experiments (Portugal [50]; Spain [6, 48]; England [4]; Germany [34]; Switzerland [12]; Italy [47]). However, the activity of geographically distant populations within the same species can differ due to their local adaptations [51–53]. It is thus very important to monitor the activity of the forensically important blowflies locally. In our dataset, there was only one species with occurrence in each month—*C. vicina*. It is a species well adapted for cold weather and during the winter season, it is often the only species found on the bodies [34]. In southern parts of Europe, it can be missing during the warm part of the year due to its upper developmental threshold of 30 °C [47, 50]. *Calliphora vomitoria* was active during the whole year except February. Both species are thermophobic with supercooling points (SCP) of − 8 °C (*C. vicina*) and – 11 °C (*C. vomitoria*) for overwintering adult flies [39]. The SCP is higher for *C. vicina*; however, due to its preference for urban habitats, it can stay active even in the coldest winters with many opportunities for warm refuges [4]. The occurrence of all other species was focused to warm summer and autumn months. Such results are supported also by [4, 12, 34, 48]. Thermophilous species of genus Lucilia were mostly present in August with the highest activity from May to October. Only *L. sericata* occurred also during the cold months of February and November; however, all of these cases were located indoors. Interestingly, live pupae were found in the case from February 1997 and the PMI_min_ was calculated to be 63 days, which would mean an oviposition at the end of December. Even though *L. sericata* is considered to be a “summer” species, it can occasionally be active even during the winter months when it seeks the warmth of human settlements. Similar to *C. vicina*, this species is also related to urban areas. All species except *C. vicina* and *C. vomitoria* were the most active during August; however, this is likely supported by the fact that August was the busiest month for the police investigators (Fig. 3). *Chrysomya albiceps* and *P. terraenovae* were irregularly distributed during the year with a peak of their activity in summer.

There was, however, a case with living pupae of *P. terraenovae* found in December 1997 in the forest. The PMI_min_ for this case was estimated to be 105 days, which would mean oviposition at the beginning of September. Some living pupae of *C. albiceps* were also found during cold months of February and April 1996 in forested areas. The PMI_min_ was calculated to be more than 6 months for both cases, which means that pupae of *C. albiceps* could be able to overwinter and survive very low temperatures. While *P. terraenovae* is considered a cold-tolerating species [20, 54], *C. albiceps* is a strictly thermophilous species [41, 49, 55]; therefore, such findings are an interesting observation. *Phormia regina* was found only in May, August and September, but the seasonal activity cannot be determined just from 7 occurrences.

### The study limitations

Similar to other studies evaluating insect data from real cases, it is important to note that they do not represent the results of a planned ecological study and while such a dataset has certain advantages, we have to be also aware of its disadvantages [19]. One of the most important differences between data collected during carefully designed experiments and real case scenarios is that we will never be able to retrieve the same amount of replications out of the latter. For example, in the presented dataset 61 % of cases were found indoor, while only 39% were outdoor. This is a typical scenario as most cases of legal investigations are conducted on bodies found inside [3]. In this article, we also decided to analyze the habitat preferences of the focal species. Due to the majority of cases being located inside, the most numerous habitat types in our dataset was “apartment” with 88 cases, while only 5 bodies were found in parks (Table 2). The same applies for the altitudinal distribution as most of the cases (77) were between 0 and 500 m, while there was only 1 case between 2000 and 2750 m (Table 3). Due to the discrepancies between these variables, we could not compare them equally. Other not-tested variables as interspecific competition or potential presence and influence of xenobiotics were not taken into consideration.

**Table 3:** The number of real case investigations for each altitudinal range during 1993–2007

Altitude range	No. of cases	Percentage
0–500	77	53.10
500–750	46	31.72
750–1000	9	6.21
1000–1250	8	5.52
1250–1500	1	0.69
1500–1750	2	1.38
1750–2000	1	0.69
2000–2750	1	0.69
**Total**	**145**	**100**

## Conclusion

The analyses of 160 real case investigations from Switzerland brought some new and interesting information about the spatio-temporal distribution of blowflies, while confirming some general expectations of their ecological requirements. The results are of a great importance for forensic entomologists and insect ecologists as they can directly help with the PMI_min_ estimation and are easily comparable with similar data from all around the world. Indeed, the fact that the dataset consisted of blowflies collected during real case investigations increases the validity of the results as the bias of studies trying to simulate real cases is omitted here. Our study compared the Swiss dataset with a similar dataset from Germany and found unexpected differences in species composition and occurrences of certain species. Such differences stem from an intricate interplay between large-scale and local-scale processes [56] as well as colonization history [57] and they only underline the importance of knowing the local populations of necrophagous insects by a forensic entomologist. Every forensic entomologist should be aware of the spatio-temporal and altitudinal distribution of necrophagous insects in the area of their activity as it significantly precises the PMI_min_ estimation as well as other predictions and analyses forensic entomologist can face. From an ecological point of view, it is interesting to study the distribution of the species in Europe, their local adaptations, and reactions to global warming (latitudinal/altitudinal shifts). In order to obtain such results, we recommend monitoring the local populations of blowflies in and around Lausanne further and comparing recent observations with this dataset.
